# An investigation into the cutting efficiency of a novel degradable glass as an alternative to alumina powder in air abrasion cutting of enamel

**DOI:** 10.1007/s00784-021-04307-7

**Published:** 2021-11-25

**Authors:** Zoi Kotsanidou, Lifong Zou, Robert Hill, Tomasz Janicki

**Affiliations:** 1 Academisch Centrum Tandheelkunde Amsterdam (ACTA), Gustav Mahlerlaan 3004, 1081 LA Amsterdam, Netherlands; 2 Barts and The London School of Medicine and Dentistry, Queen Mary University of London (QMUL), Turner Street, London, E1 2AD UK; 3 Institute of Dentistry, Dental Physical Sciences Unit, 2nd Fl. Francis Bancroft Building, Mile End, London, E1 4NS UK

**Keywords:** Air abrasion, Aluminum oxide, Degradable glass, Alternative materials, Zinc glasses, Alumina powder

## Abstract

**Objectives:**

To develop and test the cutting efficiency of a novel degradable glass as an alternative media to alumina powder for air abrasion.

**Materials and methods:**

A zinc-based glass (QMZK2) was designed, produced, and evaluated with a multi-modality imaging analysis. The glass dissolution study was carried out in three acids, using ICP-OES (inductively coupled plasma optical emission spectroscopy) at 5 different time points: 2.5, 5, 10, 60, and 240 min. The cutting efficiency of both materials was tested under the same parameters on slabs of elephant enamel. A stained fissure of a molar tooth was air abraded with the glass and evaluated with X-ray micro-tomography before and after air abrasion.

**Results:**

The particle size distribution of the glass was similar to that of alumina 53 µm but with a slightly greater dispersion of particle size. The shape of the particles was angular, appropriate for cutting purposes. The dissolution study showed that the glass dissolved rapidly in acidic conditions at all time points. Between the two variables, pressure and powder flow, pressure was found to influence the cutting speed to a greater extent than powder flow.

**Conclusions:**

Alumina powder was found to perform significantly better in 4 of the 9 conditions tested on elephant enamel, QMZK2 in one, and no significant differences were found for the rest of the 4 conditions. The QMZK2 seems to offer promising results as an alternative material to alumina.

Clinical relevance.

QMZK2 glass has the potential for replacing aluminum oxide as a degradable material in air abrasion technology.

## Introduction

Air abrasion is clinically used for tooth structure removal using high-speed stream of abrasive particles [1, 2]. It has an advantage of causing much less subsurface cracking compared to rotary instruments and lasers. Clinically, air abrasion can be used to remove carious enamel and dentine; however, gross, soft caries will not be removed effectively by air abrasion because the cutting efficiency depends on the hardness both of the cutting material and the substrate [3, 4]. In view of the fact that aluminum oxide is a hard material (2100 Knoop Hardness), it removes hard substrates better than soft ones, such as carious dentine. To overcome this problem, several researchers tested alternative cutting materials, like the bioactive glass 45S5/Sylc ®, with an attempt to selectively remove carious enamel and dentine [5–7]. The authors presented some promising results, but the methods used were semi-quantitative, the times achieved were much longer compared to alumina powder, and the authors suggested the use of 45S5/Sylc® in specific clinical situations like stained and carious fissures. Bioactive glasses (BAG) are calcium phosphosilicates that can dissolve in physiological solutions to release calcium and phosphate and to form hydiroxycarbonated apatite, which is similar to tooth mineral [8]. Milly reported that BAG powder is more efficient than alumina in the selective removal of resin composite and may be recommended as a method of repairing and removal of defective resin composite restorations, allowing to preserve sound enamel [9]. The combined use of alumina and bioactive glass-45S5 showed promising cutting efficiency, similar to that of the commercially available alumina. The alumina in this combination gives hardness to the material, and 45S5 bioactive glass promotes remineralization of the tooth structure through the formation of apatite [10]. There is evidence that enamel white spot lesions can be remineralized using bioactive glass (BAG) and polyacrylic acid–modified bioactive glass powders (PAA-BAG), which was assessed by the resultant improved mechanical properties, higher phosphate content, and morphological changes within the artificial lesions [11]. It is clinically significant that pre-conditioning of white spot lesion surfaces with PAA-BAG air abrasion modifies the lesion surface physically and enhances remineralization using BAG 45S5 therapy [12].

The effectiveness of air abrasion technique can be determined by parameters, such as air pressure, tip diameter, its length, angle of the nozzle, distance between the tip of the handpiece, and the cutting object as well as abrasive particle size [13]. The sizes of alumina particles currently in use are 29 µm and 53 µm (nominal). Both particle sizes exceed the lower limit of exposure of 10 µm set by the FDA (Food and Drug Administration). Banerjee et al*.* studied dentine surfaces after air abrasion using SEM and observed particles sizes between 2 µm and 15 µm, which indirectly suggested a possibility that particles smaller than 10 µm were present after the impact on the tooth surface in the human body causing potential toxic reactions [14].

The toxicity of aluminum oxide dust in sizes smaller than 10 µm was well documented in the literature, and toxic reactions include typical bronchopneumonia, chronic granulomatous reactions, patchy atelectasis, and emphysematous changes in rabbit’s lungs [15, 16]. In view of the above, the aim of the present study was to develop a glass as an abrasion media that removes tooth structure in times comparable to alumina but dissolves in acidic conditions, such as would be present if the glass particles are dispersed in the human body and attacked by the cells of the defense system. The null hypothesis investigated was that air abrasion with zinc-based glass can cut cavities in teeth as efficiently as alumina.

## Materials and methods

### Glass synthesis

The glass chosen for this study QMZK2 was designed to be acid soluble via the acid hydrolysis of Zn–O-Si bonds of the glass network was a similar fashion to the acid hydrolysis of Al–O–Si bonds of glasses used to form glass ionomer cements. The glass contained zinc, fluoride, magnesium, and two alkali earth oxides (CaO and SrO). The composition of the glass is listed in Table 1.

**Table 1:** Composition of QMZK2 glass (mol %)

	SiO_2_	CaO	CaF_2_	SrO	SrF_2_	MgO	ZnO
	47.32%	5.21%	5.52%	7.96%	2.96%	1.12%	30%

The glass was prepared using the melt-quench route. Correct amounts of SiO_2_, CaO, CaF_2_, SrO, SrF_2_, MgO, and ZnO (Prince Minerals LTD. Staffordshire, UK) were mixed and produced 8 batches of 200 g (± 0.01 g) each, then melted in a platinum rhodium crucible for 1.25 h at 1480 °C in an electric furnace (Lenton EHF 17/3 Sheffield UK). The glass of each batch was then rapidly quenched into cold water, washed with ethanol to accelerate the drying process, and placed in a Harvard LTE drying cabinet at 50 °C for 40 min. The total weight of glass frit obtained at the end of the glass making process was 1211 g.

From the 1211 g of glass frit, 1050 g was divided into 7 batches of 150 g each, and milled for 1 min using a vibratory percussion mill (Glen Creston Gyro-Mill, Middlesex, UK) with the objective of obtaining large glass particles. The sieving process was next performed with two sieves, 38 µm and 80 µm; the desired fraction was the powder that remained on top of the 38-µm sieve and would contain glass particles above 38 µm and below 80 µm. The sieving process took place in two stages: first, five batches of 200 g of QMZK2 were sieved for 10 min. Because the fraction above 80 µm was too large, more than half of the total powder, this fraction was re-milled for another minute. Then, the re-milled powder along with the fraction between 38 and 80 µm were sieved again for 30 min in batches of 180 g. The total amount of the powder between 38 and 80 µm obtained was 336 g.

### Particle size analysis and imaging

The glass powder between 38 and 80 µm and alumina powder 53 µm (nominal size) were analyzed with the laser diffraction technique (Mastersizer/E, Malvern Instruments, UK). The medium used was deionized water and the mixture was pumped continuously; ultrasound was also applied to the system to prevent formation of agglomerates and ensure that single, separated particles are detected.

QMZK2 particles and alumina particles (29 µm and 53 µm) were mounted on stubs via electrically conductive adhesive carbon tapes and were examined under a scanning electron microscope with a magnification of 200 × (Zeiss, Evo/MA10, Germany).

### The degradation experiment

The dissolution of QMZK2 glass particles was studied in three acidic solutions: acetic acid 6%, phosphoric acid 37%, and citric acid 6%. One liter of each solution was prepared and 0.075 g of QMZK2 was immersed in 50 ml of each solution for five different time points: 2.5 min, 5 min, 10 min, 60 min, and 4 h. The solutions with the glass were kept in a shaking incubator (IKA KS 4000ic Control Orbital Shaking Incubator) at 37 °C until the required time points, and they were subsequently vacuum-filtered with a medium porosity filter (5-µm particle retention, VWR International). The solutions were then collected in Falcon tubes. The samples were prepared with a 1:4 dilution for phosphoric and citric acid (2.5 ml of solution, 7.4 ml water, and 0.1 ml of 1% nitric acid). For acetic acid, a 1:20 dilution was followed (0.5 ml of the sample, 0.1 ml of 1% nitric acid, and 9.4 ml of water). Sample-based standard solutions were prepared for Ca, Sr, Si, Mg, and Zn and were used as standards for analysis with ICP-OES technique. Ultrapure water was used as a blank.

### Air abrasion study

The air abrasion machine used was the Aquacut Quattro model (Velopex International, UK). The machine was operated with an output pressure between 0 and 7 bars; the pressure was controlled manually; and the pressure gauge displayed 3 grades, *A*, *B*, and *C* representing pressures of 2–3.5 bars, 4–5 bars, and 6–7 bars, respectively.

A pressure gauge was attached to the machine to measure the exact pressure at any position of the switch. The powder flow control was also manual with a range of 1 to 5 and the maximum flow rate is + 70 l/min. The fluid volume control was manual and was kept fixed throughout all air-abrasion experiments. The machine can be loaded with up to two cartridges of powder and the handpiece was removable for sterilization with a nozzle diameter of 0.6 mm.

In this study, enamel slabs taken from molar teeth from an Indian elephant were used. This was to avoid the variability of hardness of human enamel due to different concentrations of fluoride in the surface, plus the fact that for quantitative measurements flat enamel was a more appropriate substrate. The microstructure of elephant enamel has been extensively studied by Boyde [17], who investigated the enamel prism pattern and crystallite orientation of 20 mammalian species belonging to nine orders (Cetacea, Insectivora, Chiroptera, Sirenia, Ungulata, Marsupialia, Proboscidea, Carnivora, and Primates) and found that the enamel prisms of the animals that belong to the orders Proboscidea [elephants] and Carnivora demonstrate the same keyhole pattern of arrangement as human enamel. In contrast, all other orders exhibit significant differences in the pattern of arrangement of the enamel prisms. The elephant molar slabs were ground and polished with abrasive papers of grit 180, 400, 500, 1000, and 2400 to create flat surfaces of enamel. Prior to the experiment, the enamel slabs were kept in a freezer at − 57 °C to avoid deterioration. A total of twelve slabs were polished; six slabs were used for cutting with QMZK2 and six slabs for cutting with alumina particles (53 µm). There were prepared six cavities per slab with distribution of two rows of three cavities each. Therefore, 36 holes were made with QMZK2 and 36 with alumina 53 µm. The three different pressures (2, 4, and 7 bars) and powder flows (2, 3, and 5 units) were tested. For each combination of the variables, four cavities were drilled. Both the enamel slabs and the handpiece were stabilized so that the handpiece was perpendicular to the flat enamel surface with a distance of 0.9 mm between them, the thickness of a microscope slide. Both materials were tested for 10 s using an electronic stopwatch. A new tip was used before the testing of each material and the machine was run for 1 min before starting the measurements to ensure that all residual particles in the hose were disposed of.

A human upper first permanent molar with a stained fissure was subjected to air abrasion with QMZK2 for as long as necessary to remove the stains from the occlusal fissures. The molar tooth was scanned with x-ray micro-tomography (µCT 40 desk-top micro CT scanner, Scanco Medical) prior and following air abrasion and the two images were subtracted using the Tomview software. The total time to remove the stain was 1 min, measured with an electronic stopwatch. The settings under which the molar was air-abraded were chosen according to the conditions under which we had the quickest cutting through the microscope slide at the first experiment (6 bar pressure and 5 powder flow).

### Analysis of the air-abraded elephant enamel

The elephant enamel surfaces were digitized by a contact profilometer (Renishaw Incise TM, UK) before the air abrasion to obtain a reference surface for each slab. The diameter of the probe tip was 1 mm. The enamel surfaces were subjected to the same method after air abrasion, and the homologous surfaces were subsequently subtracted using the Cloud software. The depth of each hole was then recorded as the deepest point detected in each cavity of the subtracted image.

### Statistical analysis

The results of this study were analyzed using the statistical package for the Social Sciences software (SPSS Inc., IBM, Chicago, IL, USA). A *t*-test was carried out for each set of quartets of values for glass and alumina obtained under the same conditions, and the level of significance was set at *P* < 0.05.

## Results

### Particle size analysis

The particle size analysis of the QMZK2 glass powder between 38 and 80 µm showed the distribution mean values: *D*50 = 49.53 µm, *D*10 = 2.47 µm, and *D*90 = 74.58 µm. Therefore, 50% of the particles were larger than 49.53 µm, 10% of the particles were smaller than 2.47 µm, and 90% of the particles were smaller 74.58 µm.

The particle size distribution of alumina powder 53 µm showed the mean values of the distribution: *D*50 = 64 µm, *D*10 = 36 µm, and *D*90 = 77 µm. Fifty percent of the particles were larger than 64 µm, 10% of the particles were smaller than 36 µm, and 90% were smaller than 77 µm.

### Scanning electron microscopy

**Fig. 1:**
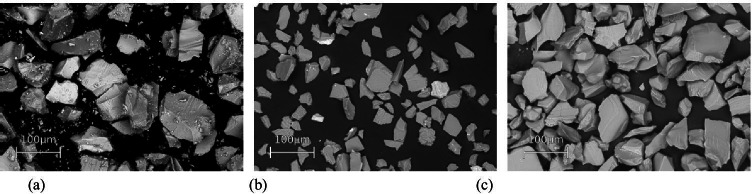
View of scanning microscope images of **a** glass particles between 28 and 80 µm, **b** alumina particles 53 µm, **c** alumina particles 29 µm

Images of QMZK2 glass particles between 28 and 80 µm, alumina particles 53 µm, and alumina particles 29 µm taken with scanning electron microscopy were presented in Fig. 1a, b, and c, respectively. In Fig. 1a, small particles sticking on the surface of larger glass particles could be clearly seen.

### The degradation experiments

The dissolution of QMZK2 glass particles was studied in three acids, phosphoric acid 37%, citric acid 6%, and acetic acid 6%. The pH of the solutions was approximately 1 for phosphoric acid and 4 for both citric and acetic acids. The percentage concentrations of dissolved Mg^2+^, Sr^2+^, Zn^2+^, Si^2+^, and Ca^2+^ in phosphoric acid after 2.5, 5, 10, 60, and 240 min and corresponding graphs are presented in Table 2 and Fig. 3a, respectively. Similarly, the percentage of dissolution in citric acid and the corresponding graph are presented in Table 2 and Fig. 3b, respectively. The percentage of dissolution in acetic acid and the corresponding graph are presented in Table 2 and Fig. 3c, respectively.

**Table 2:** Percentage dissolution in phosphoric, citric, and acetic acid

Time (min)		2.5	5	10	60	240
Phosphoric acid	Mg	60.63	75.71	75.78	87.53	86.57
	Sr	37.10	36.35	49.95	58.90	57.42
	Zn	53.01	51.08	70.90	82.94	81.33
	Si	3.65	2.49	4.67	3.33	2.58
	Ca	37.49	47.05	49.51	58.02	56.99
Citric acid	Mg	50.21	60.96	66.25	85.00	77.62
	Sr	23.04	29.73	35.39	43.06	40.44
	Zn	33.24	43.68	51.73	65.07	61.09
	Si	20.14	23.89	26.26	27.25	24.89
	Ca	17.19	25.75	31.68	41.69	38.46
Acetic acid	Mg	-	-	-	-	-
	Sr	13.15	14.45	17.60	33.19	29.87
	Zn	18.16	19.97	24.89	46.81	42.38
	Si	9.69	10.56	12.60	20.77	16.11
	Ca	17.77	19.14	21.72	36.08	32.26

### Cutting efficiency test

This test was using air abrasion on elephant enamel. Tables 3 presents the cutting depth (mm) in relation to powder flow and pressure 2, 4, and 7 bars for glass QMZK2 and alumina 53 µm, respectively. Figure 3 presents the data graphically listed in Table 3.

**Table 3:** The cutting depth (mm) in relation to powder flow and pressure 2, 4, and 7 bars for glass QMZK2 and alumina 53 µm, respectively

Flow rate	Pressure (bar)/Material and cutting depth in (mm)					
	2 /QMZK2	2 /Alumina	4/ QMZK2	4/ Alumina	7/ QMZK2	7/ Alumina
**2**	0.419	0.388	0.461	0.641	0.725	0.937
**3**	0.452	0.390	0.494	0.633	0.793	0.846
**5**	0.455	0.452	0.598	0.696	0.926	0.845

For QMZK2 glass, increasing the powder flow at a fixed pressure increased the depth of the cut, whereas that was not always the case for alumina. For the pressure of 7 bars, the alumina powder had better cutting performance for the lowest powder flow. At pressures 2 and 4 bars, alumina performed the best at powder flow rate 5. At powder flows 2 and 3, the alumina cutting properties did not change significantly within the group of 2 and 4 bar pressure application.

Figure 3 presents the cutting depth (mm) with the two materials in relation to pressure at powder flow 2, 3, and 5, respectively. It was noted that changes in depth when changing the pressure were bigger than the changes that occurred when altering the powder flow at a fixed pressure. Therefore, pressure had a greater effect on the depth of the hole cut and the cutting efficiency than the powder flow.

The *p* values for each combination of conditions are displayed in Table 4. If *p* ≤ 0.05, there were statistically significant differences (*p* ≤ 0.05) in 5 combinations:

1. Pressure 2 and powder flow rate 3, with QMZK2 performing significantly better
2. Pressure 4 and all powder flows with alumina performing significantly better
3. Pressure 7 and powder flow 2 with alumina performing significantly better

**Table 4:** *p* values of each *t*-test for each set of quartets; * indicates a significant difference at α = 0.05

*p* value		Pressure (bar)		
**Powder flow rate**		**2**	**4**	**7**
	**2**	0.2002	0.0186*	0.0040*
	**3**	0.0135*	0.0009*	0.3280
	**5**	0.4745	0.0116*	0.1827

Therefore, among the nine combinations of parameters, there was one combination where QMZK2 was performing significantly better, 4 combinations where alumina was performing significantly better, and four other combinations of parameters, where there was no statistically significant difference (*p* > 0.05) between the two materials.

### Air abrasion of stained fissure

**Fig. 2:**
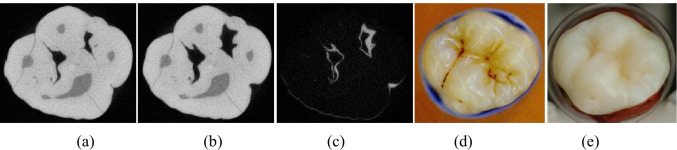
View of the molar tooth prior and following air-abrasion: **a** occlusal surface prior to air abrasion, **b** occlusal surface following air abrasion, **c** subtracted image, **d** molar tooth prior air abrasion, **e** molar tooth following air abrasion

An extracted upper first human permanent molar with a stained fissure was subjected to air abrasion until the stains of the occlusal fissures were removed and were scanned with X-ray micro-tomography before and after air abrasion. The total time to remove the stains was 1 min and the pictures of before and after as well as the subtracted images are presented in Fig. 2. The above experiment was implemented in an attempt to test the efficacy of the glass powder on a stained fissure and obtain qualitative information, rather than quantitative. To compare reliably air abrasion of stained fissure with, for instance, pumice prophylaxis and air polishing with sodium bicarbonate, it would require having identical stained fissures, which was impossible to collect.

## Discussion

### Particle size analysis and scanning electron microscopy (SEM)

In Banerjee’s study and Paolineli’s study, smaller particles of 45S5 bioglass were used, 10–40 μm and 25–32 μm, respectively, similar in size to the alumina particles [5, 6, 18]. Tan et al. used the bioactive glass (BAG) particles in the range size 38–80 µm; in comparison, larger than 29-µm alumina particles and some of the particles in this composition can be larger than 53 µm of alumina particles [19]. The bioactive glass size was modeled by Farooq, yet that study demonstrated a lower cutting efficiency of bioactive glass. In this study, multiple small particles in abrasive powder composition were used and that might have affected the cutting ability or power flow [20].

Alumina powder 53 µm has a narrower range of particle size with overall larger particles than QMZK2, which has a broader range of particle sizes with particles as fine as 3 µm. Ninety percent of the alumina particles were larger than 36 µm, whereas 90% of the QMZK2 particles were larger than 2.47 µm. The presence of the fine glass particles can be explained by the fact that smaller particles tend to stick on the surface of particles larger than 38 µm that were not able to move through the 38 µm sieve, therefore staying in the above 38-µm fraction. This can be observed in the SEM images of the QMZK2 glass.

The shape of the QMZK2 glass particles was angular with sharp edges arising from the impact fracture of the glass; this makes it appropriate for cutting purposes. In the SEM images, some fine glass particles were observed sticking onto the surface of larger glass particles confirming the wider range of glass particle sizes found during the particle size analysis. This can be observed in the SEM images of the QMZK2 glass. Additionally, the presence of the fine particles may adversely affect the cutting efficiency of the glass powder, as the particles with the most cutting potential were those of larger size (Fig.1a).

### Degradation experiment

**Fig. 3:**
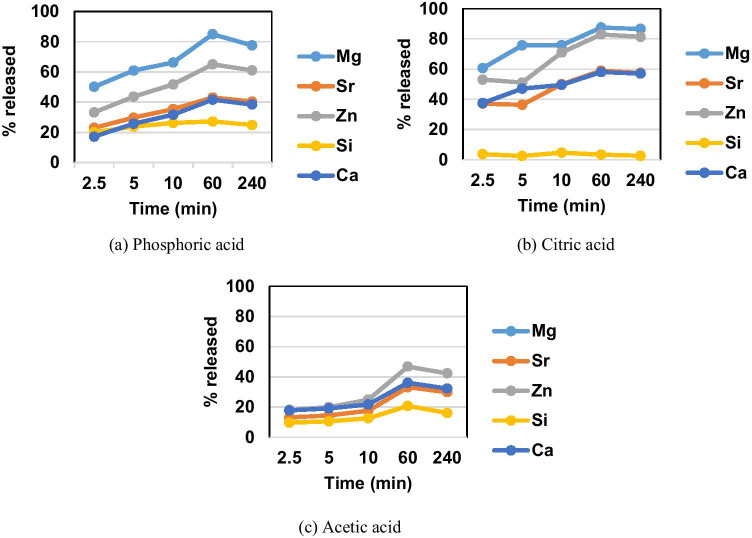
Percentage of each ion in phosphoric, citric, and acetic solution at 5 time points: 2.5, 5, 10, 60, and 240 min

Overall, phosphoric acid caused the greater dissolution of all oxides except for SiO_2_, followed by citric and acetic acid. The SiO_2_ dissolved mostly in citric acid, with acetic acid being next, leaving the strongest of the three acids and the phosphoric acid the last. With increasing time, dissolution percentages generally increased except for SiO_2_ and except for 240-min values that were slightly lower than 60-min values. This could be explained by the fact that after an hour, precipitation of salts like calcium phosphate/citrate/acetate, as well as the salts of other oxides, may take place reducing the concentration of the ions in solution. Among the oxides, MgO showed the most dissolution in phosphoric and citric acids for all the time points studied. For acetic acid, no data on the concentration of Mg^2+^ could be collected, presumably due to the low concentration of MgO in the glass (1.12%) as well as the fact that acetic acid was a weaker acid in comparison to citric and phosphoric acids leading to some release of Mg^2+^, but in levels that could not be detected. SrO and CaO exhibited similar dissolution in all acids and for almost all time points Sr^2+^ values were only slightly higher; this could be explained by the similar charge to size ratio of Ca^2+^ and Sr^2+^ that led to similar behavior of their oxides. SiO_2_ showed the least dissolution; this was presumably attributed to the fact that insoluble species like silicic acid (Si(OH)_4_)and silica gel were formed at low pH. Furthermore, the latter species along with salts that precipitate with time on the glass surface will act as a barrier to further dissolution (Fig. 3).

The dissolution of the QMZK2 glass into Tris buffer was not investigated in the present study but according to a series of dissolution studies of zinc containing glasses with similar concentrations of zinc carried out at Queen Mary University of London, the Zn^2+^ release of all the glasses into Tris buffer was negligible, in contrast to the markedly high concentrations of Zn^2+^ in acidic conditions [21, 22]. These findings suggest that zinc predominantly acts as an intermediate oxide and forms ZnO_4_ units, and Zn–O-Si bonds similarly to AlO_4_ units and Al–O–Si bonds present in ionomer glasses used for cements that can be hydrolyzed only under acidic conditions. The practical importance of this finding lies in the ability of the powder to remain stable when atmospheric moisture is present during storage, preventing a partial degradation of the powder before its use that would adversely influence its cutting properties.

### Air abrasion on elephant enamel

**Fig. 4:**
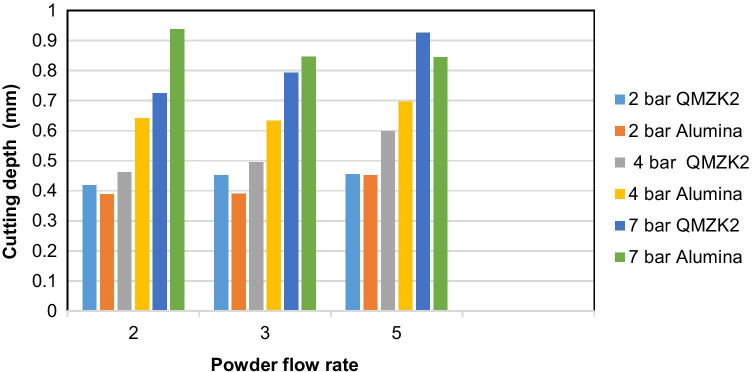
Cutting depth (mm) with the materials: QMZK2 and alumina 53 µm in relation to powder flow at pressure 2, 4, and 7 bars

The cutting process during air abrasion probably involves material removal by impact of the particles on the tooth surface which is determined by the kinetic energy of the particles which depend on both their mass and velocity plus there is an element of slurry cutting where the particles are centrifugally spun as a water slurry in deeper cavities removing more enamel towards the periphery. We could see from the above data that for QMZK2 glass, increasing the powder flow at a fixed pressure increased the depth of the cut, whereas that was not always the case for alumina. These findings confirmed the null hypothesis of this study that zinc-based glass can cut cavities in teeth as efficiently as alumina. For the pressure of 7 bars, the alumina powder had better performance for the lowest powder flow rate, and the cut depth decreases for powder flows 3 and 5 (Fig. 4). This finding was in accordance with the findings of Cook et al*.* who attempted imaging the air abrasive cutting in real time with confocal microscopy and observed a reduction in cutting rates when excessive amount of abrasive and pressure were applied onto the substrate (human premolar) [3]. The researchers observed flashes of reflected light that represented areas of “crowding” with alumina powder, momentarily disrupting the exhaust flow and lowering the cutting rates. However, better understanding of the complex mechanism of cutting through the combination of fluid and particle abrasion is needed in order to better understand the above phenomenon, as well as to why the same was not observed with the glass particles.

Little research has been carried out investigating alternative materials to alumina for air abrasion. A few authors attempted to compare the cutting efficiency of the bioactive glass 45S5 to that of the alumina mostly aiming to selectively remove carious enamel and dentine. Banerjee et al*.* compared the efficacy of alumina 29 µm and that of the bioglass 45S5 in removing enamel of both sound and carious, non-cavitated fissures scored with the ICDAS II (scores 1 and 2). The authors found that the bioglass 45S5 was consistently able to selectively remove carious enamel, but not sound enamel, whereas alumina was able to remove bulk enamel in both situations. They attributed this selectivity, to the lower hardness of the bioglass 45S5 (420 Knoop Hardness) in contrast to the much higher hardness of alumina (2100 Knoop hardness) [6]. Because sound enamel has Knoop Hardness of 390, it is to be expected that the 45S5 glass would only remove substrate that is much softer than itself, such as carious enamel, whereas alumina can easily remove the much softer sound enamel. However, the times needed for the selective removal of carious enamel with the bioglass were much higher than those needed when alumina was used and the authors suggest the use of bioglass in the specific clinical situation of stained-carious, non-cavitated fissures, rather than proposing a material that would entirely substitute for alumina in all situations. The Vickers hardness of Bioglass 45S5 reported in the literature various depending on the study: 4.5 GPa [23], 5.75 GPa [24], and 5.84 GPa [20]. For hardness measurements, glass monolithic specimen must be casted, but in this study, the glass crystallized so it was impossible to measure its hardness, but the authors would expect the glass to have a higher hardness than the sodium free bioactive glass (6.66 GPa) studied by Farooq et al.[20].

Finally, Paolinelis et al*.* used the same bioactive glass to investigate its performance on human dentine and found that bioactive glass removed both carious and sound dentine at a slower rate than alumina at equivalent pressures and feed rates, but the difference in cutting rates between carious and sound dentine was smaller for 45S5 than for alumina [5]. It is worth mentioning that both studies used semi-quantitative methods without giving exact numbers for tissue removed.

The high-phosphate fluoride containing bioactive glass have calcium to phosphate ratio of < 2.5 which was closer to the apatite stoichiometry and that glass was able to form fluorapatite, which was much more resistant to acid dissolution than hydroxyapatite. This bioactive glass can release calcium phosphate and fluoride ions simultaneously when dissolved [25, 26]. Tan et al. adopted a composition of NaOSR from Farooq et al., where fluoride was incorporated into the composition to enhance its remineralization properties while strontium was included to increase radiopacity. The researchers compared the cutting efficiency of dentine using a customized fluoride containing bioactive glass and found that NaOSR glass particles create deeper cavities than alumina despite its lower powder outflow rate predictably reduced hardness. In that study, it was proved that NaOSR was more effective than alumina in air abrasion dentine cutting [19, 20]. Farooq et al. substituted Na_2_ O for Ca O, which caused pronounced decrease in glass hardness resulting in longer cutting times by air abrasion. Low strontium content bioactive glass shows mechanical properties suitable for air abrasion application and are highly bioactive in vitro, forming apatite in Tris buffer solution within 6 h, which was significantly faster than Bioglass 45S5. Cutting times using bioactive glass were significantly longer than using the alumina 29 µm [20]. Taha et al. showed that the novel fluoride-containing QMAT3 glass was capable of enhancing enamel remineralization more effectively than bioactive glass used if the product named Sylc, promoting remineralization of white spot lesions, forming fluorapatite when in contact with physiologic-like solutions [27].

The limitation of the study was its lab-based nature which not fully represents clinical conditions where some of the air-abrasion operating parameters, like nozzle-tooth distance or nozzle angle are variable and are fully operator-dependent. The other limitation was difficulty to standardize air abrasion of stained fissure on a natural tooth and comparison of various abrasive materials as it was impossible to carry out the experiments on exactly same fissures characteristics.

## Conclusion

The null hypothesis investigated in this study was accepted. The air abrasion technique with zinc-based glass can cut cavities in teeth as effectively as alumina. The QMZK2 seems to offer promising results as an alternative material to aluminum oxide in terms of cutting speed and physical behavior when in contact with acidic environment such as would be present if glass particles enter biological tracts of the human body. Further research is needed before any clinical recommendations could be done.
